# The novel *Orshina* Rhythm in a colonial urochordate signifies the display of recurrent aging/rejuvenation sequels

**DOI:** 10.1038/s41598-023-36923-6

**Published:** 2023-06-16

**Authors:** Oshrat Ben-Hamo, Ido Izhaki, Rachel Ben-Shlomo, Baruch Rinkevich

**Affiliations:** 1 National Institute of Oceanography, Tel Shikmona, P.O. Box 9753, 3109701 Haifa, Israel; 2 Department of Evolutionary and Environmental Biology, Faculty of Natural Sciences, University of Haifa, Mount Carmel, 3498838 Haifa, Israel; 3 Department of Biology and Environment, Faculty of Natural Sciences, University of Haifa – Oranim, 36006 Tivon, Israel

**Keywords:** Ecology, Evolutionary ecology

## Abstract

When it comes to aging, some colonial invertebrates present disparate patterns from the customary aging phenomenon in unitary organisms, where a single senescence phenomenon along ontogeny culminates in their inevitable deaths. Here we studied aging processes in 81 colonies of the marine urochordate *Botryllus schlosseri* each followed from birth to death (over 720 days). The colonies were divided between three life history strategies, each distinct from the others based on the presence/absence of colonial fission: NF (no fission), FA (fission develops after the colony reaches maximal size), and FB (fission develops before the colony reaches maximal size). The study revealed recurring patterns in sexual reproductive statuses (hermaphroditism and male-only settings), colonial vigor, and size. These recurring patterns, collectively referred to as an *Orshina*, with one or more 'astogenic segments' on the genotype level. The combination of these segments forms the *Orshina* rhythm. Each *Orshina* segment lasts about three months (equivalent to 13 blastogenic cycles), and concludes with either the colonial death or rejuvenation, and is manipulated by absence/existing of fission events in NF/FA/FB strategies. These findings indicate that reproduction, life span, death, rejuvenation and fission events are important scheduled biological components in the constructed *Orshina* rhythm, a novel aging phenomenon.

## Introduction

The nature of life does not preclude an indefinite, long lasting life span of multicellular organisms. Some organisms may escape death by rejuvenation, such as the immortal hydrozoan *Turritopsis nutricula*^1^, or may exhibit prolonged life spans such as the Greenland shark, *Somniosus microcephalus*^2^, the black coral *Leiopathes glaberrima*^3^ and the sponge class *Hexactinellida*^4^. In fact, most multicellular organisms age through a single and directed ontogenic senescence phenomenon that culminates in their inevitable deaths.

**Figure 1:**
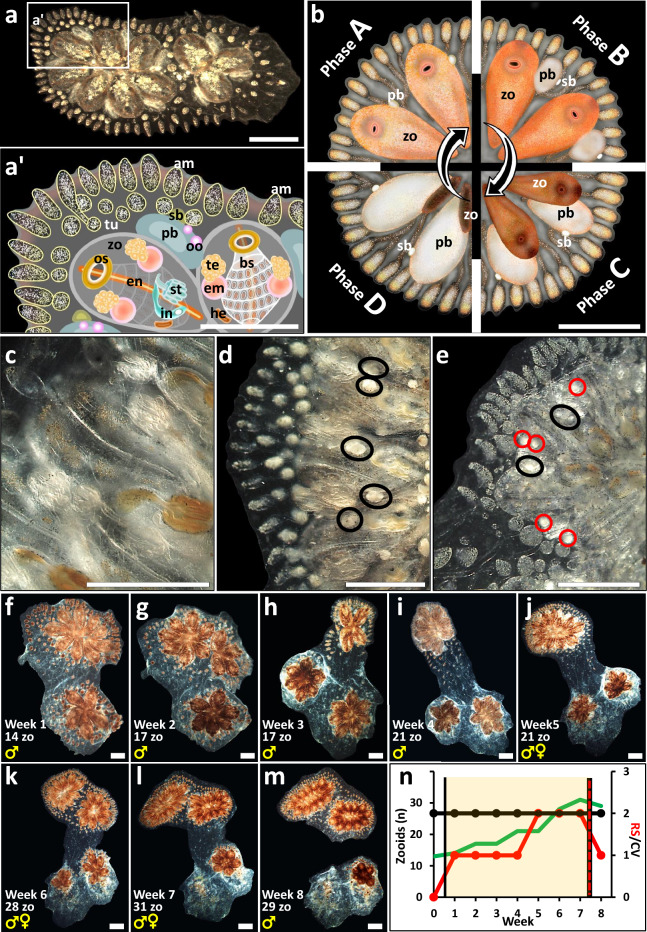
*Botryllus schlosseri* morphology and anatomy (**a**–**e**). (**a**) Mature colony composed of two flower-like shaped colonial systems, including 11 zooids. (**a’**) An illustrated magnification of (**a**) framed section, depicting the *Botryllus* colony organs. *Am* ampullae; *bs* branchial sac; *em* embryo; *en* endostyle; *he* heart; *in* intestine; *os* oral siphon; *oo* oocyte; *pb* primary bud; *st* stomach; *sb* secondary bud; *tu* tunic; *zo* zooid. (**b**) Blastogenic cycle, a weekly recurring phenomenon of life-and-death of modules, divided into four phases, A to D, *sensu*^10,42^. The cycle encompasses three co-existing generations of modules: zooids, primary buds and secondary buds. During blastogenesis, primary and secondary buds grow, but their oral siphons are not open yet as in functional zooids. At the end of each cycle (phase D or ‘takeover’) zooids are absorbed by an apoptotic wave and phagocytosis. The beginning of a new phase A is marked by the opening of the siphons of the primary set of buds. *zo* zooid, *pb* primary bud, *sb* secondary bud. (**c**–**e**). Mature zooids presenting different reproductive statuses (RS). (**c**) Sterile zooid. (**d**) Male-only zooid. (**e**) Hermaphrodite zooid. Testes are black encircled; Oocytes are red encircled. (**f**–**m**). A colony along a single ‘astogenic segment’ (an *Orshina* segment) through 8 consecutive weekly observations (**f**–**m**). During the first four weeks (**f**–**i**), the colony is male-only (RS = 1; red curve). and hermaphrodite (RS = 2) along the next three weeks (**j**–**l**). In the eighth week observed, the colony returns to a male only status (RS = 1). In this example a single fission event was observed. (**n**) A schematic illustration assembling the three colonial demographic parameters documented in observations f-m. Borders (black vertical lines) are placed according to border rules for an astogenic segment (yellow transparent square). X axis signifies the documented period (weeks). The left Y axis is for the zooid numbers (green curve) and the right Y axis for either the RS (red curve) or CV (black curve). Scale bars = 1 mm.

For aging, we studied here the model species *Botryllus schlosseri* (Fig. 1a,a’), a cosmopolitan marine colonial tunicate^5^, that has emerged as an important species in diverse biological disciplines, including aging, allorecognition, and regeneration^6–9^. In addition to whole organismal aging, *Botryllus* presents well-studied weekly rhythmic cycles of life-and-death of its functioning modules (the zooids), each termed a blastogenic cycle^10,11^ (Fig. 1b), a phenomenon considered as the ‘underwater phoenix’^12^. In each colony, three generations of modules co-exist, all genetically identical and borne asexually, altogether embedded within a gelatinous supporting matrix, the tunic (Fig. 1a’). The tunic further holds a ramified blood system connecting all modules to each other. The oldest generation of modules are the functioning zooids (Fig. 1a,b), that feed and breed. At the same time, the two younger generations, the first and secondary buds, undergo fast ontogenic development (Fig. 1a,b). As in the Phoenix, the ancient Egyptian mythical bird that ignites itself to ashes and then emerges from its own ashes utterly renewed in endless cycles, each generation of zooids in *Botryllus* colonies are consumed by a weekly apoptotic wave^10^ and replaced by the swiftly matured generation of the first buds that become the next generation of functional zooids. Thus, *B. schlosseri* represents a colonial organism with repeated mosaic-like aging at the module level and with a high regenerative power expressed by the replacement of modules that do not age in the classical sense^13^. Aging at the whole-genet level (the sum of all ramets) may also occur, and when this develops it reflects a sharp contrast to the common pathways of senescence in unitary organisms^14–16^.

**Figure 2:**
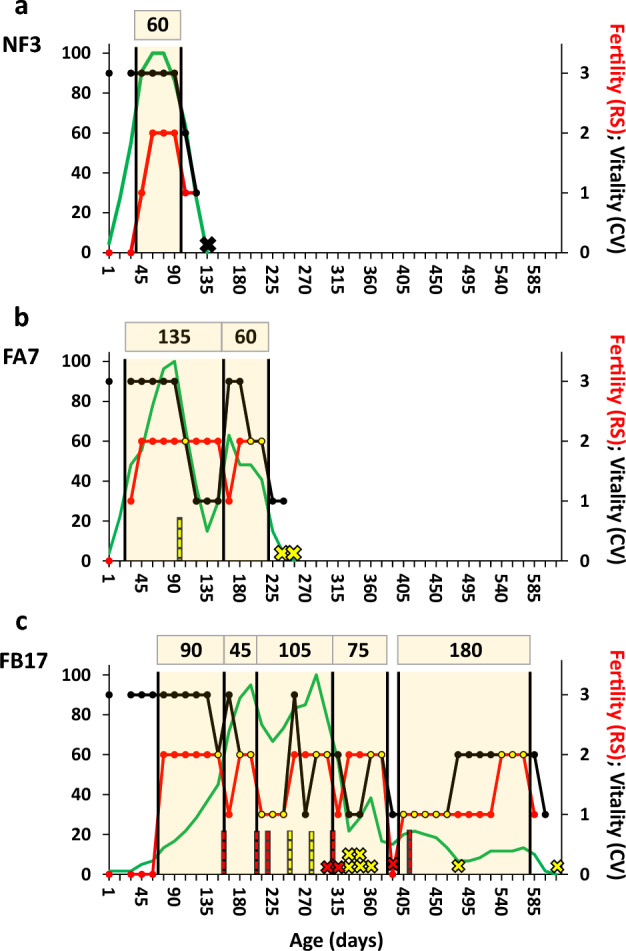
(**a**–**c**). Representative colonies that depict the ‘astogenic segments’ (yellow transparent squares) and the number of zooids, RS, and CV for each one of the three life history strategies, NF, FA and FB. X axis signifies the documented period (days), the left Y axis is for the zooid numbers (green curve) and the right Y axis for either RS (red curve) or CV (black curve). (**a**) A non-fissioned type (NF), colony NF3. The colony presents a bell-shaped growth pattern, survived for a single RS segment and started with the maximal CV. (**b**) An FA type that fission after reaching the peak size, colony FA7. The colony presents two bell-shaped growth patterns, each in a different ‘astogenic segment’ survived for two RS segments, and went through a single fission event at the age of 3 months (divided into two ramets). The genet started with maximal CV, followed by a minimal CV period and rejuvenation to a second ‘astogenic segment’ that completed by the death of the two ramets in two consecutive observations. (**c**) An FB type that fission before reaching the peak size, colony FB17. The colony presents three-four bell-shaped growth patterns, each in a different ‘astogenic segment’ survived for five RS segments and went through seven fission events (divided into ten ramets). The colony started with maximal CV, and experienced 5 or 6 rejuvenation events along life. Death events are marked with crosses. Red crosses represent deaths located adjacent to the ‘astogenic segment’ borders, yellow crosses represent deaths away from the ‘astogenic segment’ borders. Black cross represents a dead colony in NF. Fission events are marked with vertical dashed lines. Red dashed lines represent fissions adjacent to ‘astogenic segment’ borders. Yellow dashed lines represent fissions away from the ‘astogenic segment’ borders.

*Botryllus schlosseri* colonies are further known to present two major life-history strategies, semelparity and iteroparity, each signified by coevolved sets of trade-offs^17^. A recent study that followed *B. schlosseri* colonies from birth to death under relaxed laboratory conditions^18^ has elucidated three additional, yet distinct, life-history strategies, each disparate from the others, based on the presence/absence of colonial fission into two or more independent living ramets and fission properties: NF (no fission along the life span), FA (fission develops after the colony reaches its maximal number of zooids), and FB (fission develops before the colony reaches its maximal number of zooids (Fig. 2a–c).

Studying in depth various demographic traits along the life spans of *Botryllus* colonies of the 81 genotypes studied in the former study^18^, we unveiled here a second phoenix-like phenomenon, developing on the colonial level. We noticed that sexual reproductive status (hermaphroditism, male only and sterile settings), colonial vigorousness and colonial sizes, altogether represent coinciding and repeated rhythms of astogenic segments at the whole-genet level. Each astogenic segments is termed as *Orshina*, symbolizing the immortal Jewish mystical bird^19^ and the sum of all segments as the *Orshina* rhythm. The *Orshina* rhythm is typified by scheduled orchestrated morphological phenomena of colonial degradation, rejuvenation, reproduction, fission, and death of ramets.

## Materials and methods

### Animals

Colonies of *Botryllus schlosseri* were reared and kept under constant temperature (20 °C), light/dark regimen (12:12 h) in 24 L standing seawater plastic tanks, and colonies in each aquarium were daily fed with a 10 mL mixture of invertebrate food containing rotifers and microalgae. The observations were carried out at the National Institute of Oceanography (Haifa, Israel) facility, as described^20^. Colonies were followed from birth to death and observations were performed every 15 ± 5 days starting from the age of 1 month, under Nikon stereo microscope (SMZ 1000). The documented parameters included:

(a) Number of zooids: Active zooids were counted during each observation for each genet, e.g., following fissions events, the total number of zooids from all ramets were combined; (b) Colonial Vigorousness (CV): A semi-qualitative score, averaging the morphological statuses (ranging 1–3) for each one of the three major colonial compartments, the zooids/buds, the peripheral ampullae, and the tunic. CV scores ranged from 1 to 3, where 3 signifies most vigorous, 2 is an intermediate status and 1 is the lowest (Suppl. Fig. 4); (c) Reproductive Status (RS): Colonies of *B. schlosseri* present alternate sexual status (sterile, male only, hermaphrodites) during their life span. At onset, colonies are sterile, then male gonads appear first during ontogeny, and female gonads appear in the following 2–4 blastogenic cycles (Fig. 1c–e). This hermaphroditic phase is followed by periods of sexual sterility and/or only male status^20,21^. The RS is a genotype specific status which is varied between different colonies under the same environmental settings^21^. RS is a semi-quantitative score, where grade 0 represents ‘sexual sterility’ status, grade 1 for ‘only male’ colonies, and grade 2 for ‘hermaphrodite status’, irrespective to the number of female/male gonads per zooid/bud; (d) Colony fission: Colonial fission (Fig. 1f–n) is an astogenic process ending in the splitting of a colony into two or more ramets, a well-documented process in *B. schlosseri* colonies^21^. As fission gradually develops (few days to weeks from commence), the exact date of fission is determined from the first documented cut-off of blood vessels between splitting ramets, even though they may still be physically connected by degraded tunic matrix.

### Statistical analyses

Spearman’s correlation coefficient was calculated between the three documented traits (zooid numbers, CV and RS) for each colony. Then the average and standard deviation of Spearman correlation coefficients for all colonies of the same type of life history strategy (NF, FA, FB) were calculated. A one sample T test was used to examine if each average correlation between each couple of parameters for each life history type was significantly different from zero. For fissioned colonies, Chi square tests were designed to evaluate: (a) Non-random death of ramets, performed on simple cases of fission events. (b) Deaths of ramets and their associations with astogenic parameters. (c) Fission events, associations with astogenic borders. Chi-square test analyses were performed using Microsoft Excel program. Examining the possibility that death events are dependent samples, A Wilcoxon signed-rank test was conducted using SPSS program and was designed to further evaluate the proximity of ramets' death to astogenic borders. When applicable, data is presented as means ± standard deviation.

## Results

### General

**Figure 3:**
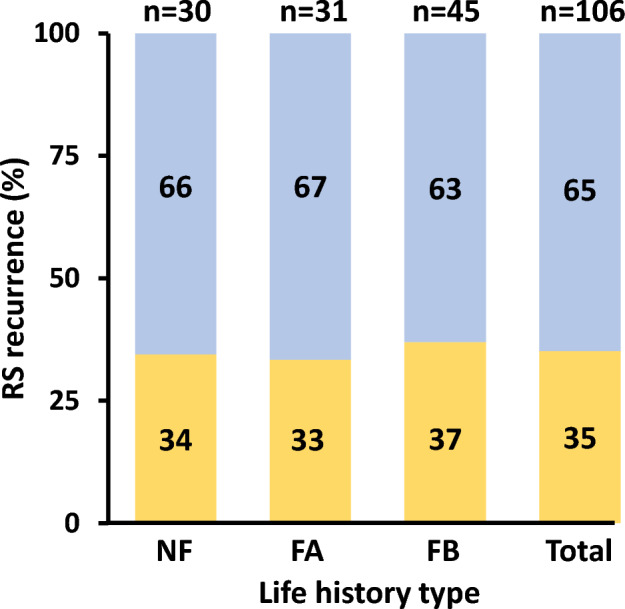
Male-only status (yellow) and hermaphroditic status (blue) distributions (%) in typical ‘astogenic segments’ of the three life-history types (NF, FA and FB) and the average summary (total) for *B. Schlosseri* colonies. The number of total segments studied for each life-history type are shown above each column.

The documented parameters, number of zooids, RS, and CV for each of the 81 colonies (NF = 35; FA = 23; FB = 23) were organized individually (examples in Fig. 2a–c; All graphs in Suppl. Figs. 1–3). Focusing first on RS, we noticed repeated cycles of reproductive statuses along the colonies’ life spans, where the first wave starts with a sterile period. A male-only status emerges later, trailed by a period of hermaphroditic status. The length of the male-only period to hermaphrodite period is regarded as a single segment. The sterile period at the beginning of life was excluded from segments delineation, since it was documented in only 8 short-term midlife periods (in colonies NF30, NF32, FA22, FB2, FB5, FB10, FB17, FB20; Suppl. Fig. 1–3). Each of the RS cycles (hereby referred to astogenic segment) was delineated (vertical lines in Fig. 2a–c). The average length of each astogenic segment was found to be 89 ± 45 days, based on a total of 142 segments from 70 colonies in total. Figure 2a–c provide visual representations of the NF, FA, and FB life history strategies as examples. Gradual differences between segment lengths among the life history strategies, albeit non-significant, were documented, with NF showed the shortest length (75 ± 31, n = 30 segments), FA an intermediate length (86 ± 38, n = 42 segments), and FB presented the longest segment length (98 ± 53, n = 70 segments). Hermaphroditism spanned about 2/3 of the astogenic segment length in all, NF, FA and FB colonies. Interestingly, the three types presented constant and almost fixed RS ratios of males vs. hermaphrodites (Fig. 3and Suppl. Table 2).

A high correlation was found between the number of astogenic segments and life span lengths (Pearson correlation coefficient 0.8; n = 77; *p* < 0.05). The NF strategy exhibits 1.3 ± 0.6 astogenic segments (n = 32) along the colonies’ life spans, followed by the FA strategy (2.2 ± 1.5 segments; n = 22) and the FB strategy (3.2 ± 1.3 segments; n = 23). Pairwise Spearman’s correlation coefficients were calculated for each pair of parameters throughout the life of each colony, summing up to 50 observations per pair (no. of zooids vs. RS; no. of zooids vs. CV; RS vs. CV; Suppl. Table 1a), followed by one-sample t-test analyses to test the significance of the average correlations for each pair of parameters in each life history strategy. The results revealed significant correlations, mostly positive, for most pairs (Suppl. Table 1b). In the NF all Zooids-RS, Zooids-CV and RS-CV correlations were significant (*p* < 0.005), while in the FA and FB, two pairs were significant: Zooids-RS, Zooids-CV, and Zooids-RS, RS-CV, respectively (Suppl. Table 1b). In the three life strategies, zooids vs. RS and zooids vs. CV showed positive correlations, while the RS vs. CV pair showed a negative correlation, suggesting antagonistic expressions (Suppl. Table 1a). In the four longest-living colonies (FB 20–23) with life spans of 623, 458, 471 and 726 days, respectively, the three pairwise combination tests were significant (*p* < 0.05), except the pair ‘zooids vs. CV’ in colony FB21.

### Ramet’s death and fission are synchronized with the astogenic segments

**Figure 4:**
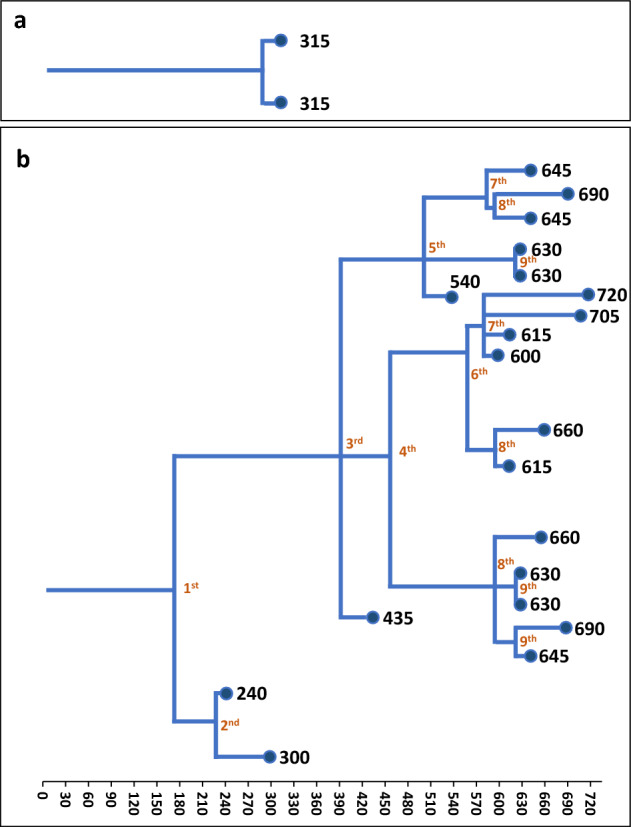
Fission sequences in *B. Schlosseri* colonies. (**a**) A colony that fissioned only once (FA16). (**b**) A colony (colony FB3) that went through 9 fission events, creating 20 ramets. Black numbers depict life-long periods (days) for each ramet. Orange numbers show chronological sequence of the fission events.

We then focused on fission events and ramets’ deaths. FA and FB colonies underwent fission events, forming naturally detached ramets that survived for a few days to prolonged periods of time (deaths are marked with crosses in Fig. 2a–c), resulting in either simple cases of a single fission event with just two ramets (Fig. 4a) or complex cases of multiple ramets per genet (Fig. 4b). For statistical analyses on ramets’ life spans, we focused only on colonies that underwent a single fission event (n = 14 cases; FA and FB types; Suppl. Table 3). Of these, 50% of the paired ramets died simultaneously and non-randomly, indicating that both ramets/genet died together (observed as dead in the same or consecutive observation). Statistical analyses indicated a significant likelihood of the ramets to die at the same time (Chi-square test, *p* < 0.001, n = 14, df = 1; Suppl. Table 4).

Observations further revealed that ramets’ deaths were aligned, in many cases, with the borders of the astogenic segments. An example with two ramets that died adjacent to the segment borders is depicted in Fig. 2c (red crosses). As colonies were observed every 15 days and as borders are delineated between observations, a death associated with the segment border was assigned to ramets dying within a period of 7.5 days before or after a border line. Since the segment borders cannot be delineated at the end of genet’s life span, we did not include death cases allied with the genet demise in the Chi square analyses, therefore, death cases occurring up to the midpoint of the last astogenic segment (45 days) were included. Results revealed that ramets deaths were significantly adjacent to the astogenic borders (Chi square, *p* < 0.0001, df = 1, n = 126 ramets deaths in 29 genotypes with defined segments; Suppl. Table 5). Wilcoxon sign test further confirmed non-random increased deaths at the borders of the segments (*p* < 0.0001, z = − 3.49, n = 126 ramets deaths in 29 genets), signifying that ramets’ deaths are matched with the outskirts of the segments.

Observations further revealed many cases where fission events were aligned with the borders of segments. An example with five fission events that occurred adjacent to the segment borders is depicted in Fig. 2c (dashed red lines). Since both borders and fissions are delineated between observations, a fission event associated with a border was assigned to ramets dying up to 15 days before or after the border line. As above, we included fission events occurring within the central portion of the last astogenic segment (45 days). Results revealed that fission events were significantly adjacent to the astogenic borders (Chi square test, *p* < 0.0001, df = 1, n = 165 fission events in 41 genets; Suppl. Table 6). The Wilcoxon sign test did not reveal a significant relationship (*p* > 0.05) between the astogenic segment outskirts and the occurrence of fission events.

### The astogenic rhythm

**Figure 5:**
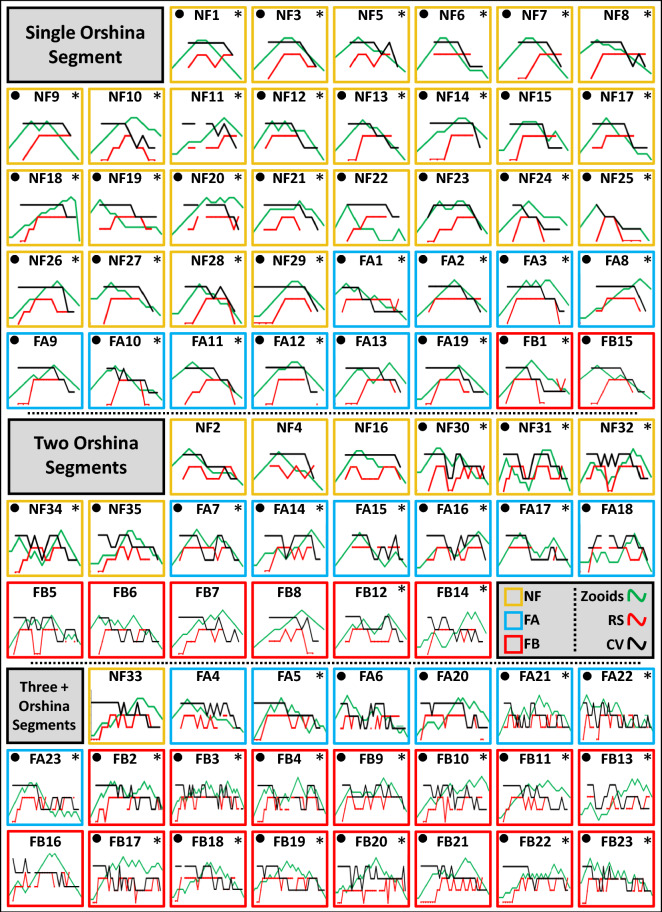
All studied genotypes (n = 81) that were followed from birth to death in accordance with the three life history types (NF = yellow frames; FA = blue frames; FB = red frames). Three demographic features are depicted for each genotype: growth trends of zooids (green curves), RS (red curves) and CV (black curves). The genotypes are divided into three groups: 1. Colonies with a single astogenic segment 2. Colonies with two astogenic segments; 3. Colonies with three or more astogenic segments. Genotypes where the number of segments matched number of zooids peaks are marked with asterisks. genotypes where the number of segments matched with the number of CV peaks are marked with black circles.

To assess the rhythmic nature of astogenic segments along the whole life spans of the colonies, we developed a method that enables the transformation of observed growth rates (changes in the number of zooids between two observations) into ’growth trends’ (measured as positive [+] or negative [−] change in zooid numbers between two observations). This allows the comparison of growth between colonies, regardless of the number of zooids. For this purpose, the data presented in the individual growth charts (Suppl. Figs. 1–3) were modified as followed: A numeral ‘1’ was assigned to the first observation of the first settled zooid. We then added the value ‘1’ every time the number of zooids at the following observation was higher than in the former observation (e.g., 2), and subtracted the value ‘1’ from the observation value where the next census revealed lower number of zooids (e.g., 0). Growth trend curves for the 81 genets (Fig. 5; green curves) were further aligned with RS and CV curves, and divided into three groups: 1. Colonies exhibiting life span of a single astogenic segment; 2. Colonies with two astogenic segments; 3. Colonies with three or more astogenic segments along their lives. For each of the 81 genotypes, the three life history types are highlighted further (NF-yellow frames; FA-blue frames; FB-red frames; Fig. 5).

Results (Fig. 5) reveal that NF colonies are more prominent in the ‘single astogenic segment’ group (60% of all genotypes), FA colonies are divided more or less equally between the three astogenic groups and FB colonies are most evident in the third astogenic group (70% of all genotypes). Using the ‘growth trends’ analyses in the group ‘single astogenic segment’, 84% of colonies further show a single peak of zooids (32/38 colonies) compared to 55% of colonies present two clear peaks (11/20 colonies) in the ‘two astogenic segments’ group, and 74% of colonies presenting several peaks (17/23 colonies) in the ‘3 + astogenic segments’ group (marked with asterisks in Fig. 5).

Analyses were then performed on Colonial Vigorousness (CV) peaks along the life span of each genotype. About 84% (32/38) of ‘single astogenic segment’ colonies present a single CV peak, 45% (9/20) of the ‘two astogenic segments’ colonies show two CV peaks and 83% (19/23) of ‘3 + astogenic segments’ colonies present several CV peaks (marked by black circles; Fig. 5). Remarkably, 60% (49/81) of the colonies show a match between the numbers of astogenic segments, the number of zooidal peaks, and the number of CV peaks, an indication of a rhythmic nature along the colonies’ life spans (marked by both asterisks and circles; Fig. 5).

### The four stages of the astogenic rhythm

**Figure 6:**
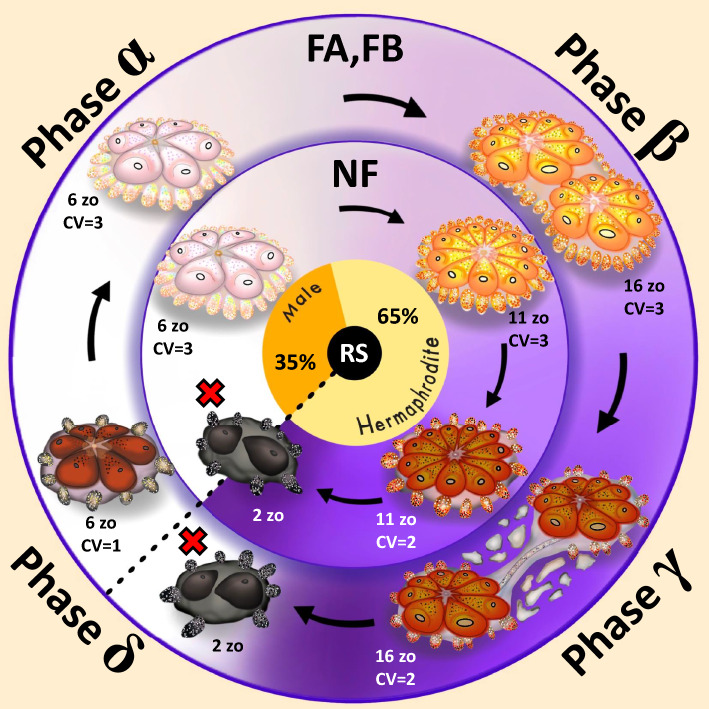
Summary of *Orshina* segment/s in non-fissioned colonies (NF, inner cycle) and in fissioned colonies (FA, FB, outer cycle). Each *Orshina* segment is divided into four phases, α through δ. In NF colonies, the rhythm repeats primarily only once, and in the fissioned colonies the rhythm usually repeats for two to three times (under lab conditions) until final death of the last ramet. The following description of the phases concerns both inner and outer cycles. The reproductive status, RS, is shown in the innermost cycle, relating to the status along all phases. Phase α: the colony presents with male-only gonads and high CV status, (CV = 3). Phase β: the developmental phase where colony’s size is greater than in phase α. Hermaphrodite status and high CV statuses are common. Phase γ: while hermaphrodite status is present, CV status may be lower than in phase β. In the fissioned types the tunic between the systems is gradually deteriorating. Phase δ: NF colonies usually die at the end of this phase. Both the death of ramets and fission events appear in this phase. Survived ramets present smaller size, lower CV, and then they go through rejuvenation at the end of this phase, enabling the ramets to carry out a new *Orshina* segment. The *Orshina* border is marked by a dashed line. Numbers of zooids present are a suggested simplification. Different colonies present different sizes, shapes, and colors. Red crosses = dead entities; zo = zooids.

Analysis of all astogenic segments in all 81 colonies studied (Figs. 2, 5 and Suppl. Figs. 1–3), revealed common morphometric features among all three life history types (NF, FA, FB), in relation to the number of zooids, RS, and CV, that are characterized by repeated astogenic segments, each divided into four developmental phases, α, β, γ, and δ (Fig. 6). In addition, during the first 35% of the length of each segment, the colony is ‘male only’, and then, for the rest 65% of the segment, it reveals the hermaphroditic state (Fig. 3; Suppl. Table 2; the first segment is delineated from the end of the juvenile sterile phase). In the astogenic segment phase α, the colony has a high CV status, presenting only male gonads. The astogenic segment phase β is a developmental state where the colony is hermaphrodite with higher CV status and represents a peak number of zooids. In astogenic segment phase γ, the colony typically presents a reduced CV status, hermaphroditic state and gradually reduced number of zooids. In the fissioned types this is also the phase where colonial fission develops. Phase δ is typified with gradually reduced CV status, reduced hermaphroditism and also with colonial fission events, when developed. The end of phase δ is the ‘turn point’ or the pivotal phase where the ramet/genet die (most NF colonies, ramets of FA/FB genotypes, or entire FA/FB genotypes) or rejuvenate into phase α of the next astogenic segment (ramets and/or whole FA/FB genotypes; (Fig. 6). Having these results (Fig. 6), each of the astogenic segments in the life span of a colony is called an ‘*Orshina* segment’ and the sum of repeating astogenic segments per a *Botryllus* genotype, is termed the ‘*Orshina* rhythm’.

## Discussion

The name *Orshina* is taken from the esoteric Jewish mysticism essays (in the Babylonian Talmud), referring to a bird that was secured in Noah’s arch. According to the legend, *Orshina* remained silent while Noah supplied food for the rest of the survived animals, and for its politeness, *Orshina* was blessed with immortal life, where every one-thousand years it degrades and then rejuvenates^19^. As a metaphor, we tagged the genet level’s life and death cycles in *Botryllus* colonies as the *Orshina* rhythm and each cycle as an *Orshina* segment (Fig. 6). Each such segment lasts approximately three months, is not linked to the weekly life and death blastogenic cycles developing at the modules level, and exists in all three *Botryllus schlosseri* life history strategies (NF: colonies that never fissioned; FA: colonies that fissioned after the maximal peak of zooids; FB: colonies that fissioned before the maximal peak of zooids^18^). Here, we further elucidated the *Orshina* rhythm, orchestrated by three traits at the colony level (expressed in different ramets and/or the whole genet), the colonial reproduction status (RS), the genet’s size (total number of zooids) and the colonial vigorousness status (CV). Each of these colonial traits portrays repeating archetypal curves over time, and all traits are further synchronized with each other, making an overall rhythm of reproduction, colonial fission, life/death, and aging/rejuvenation phenomena.

As specified, an *Orshina* rhythm is composed of one or more *Orshina* segments (each 89 ± 45 days long), each is represented by four development phases (α, β, γ and δ; Fig. 6). Regardless of the life history strategy, or the considerable variance of segments lengths, the male and the hermaphrodite phases within an *Orshina* segment occupy 35% and 65% of the *Orshina* segment length, respectively (Figs. 3,6), implying uniformity of reproductive statuses. An *Orshina* segment is also typified with a characteristic bell-shaped colonial size, where maximal size occurs at the middle of a segment.

Any *B. schlosseri* genet may exhibit several constructed aging/rejuvenation phenomena (some of them simultaneously occurring), such as semelparity (e.g. single reproductive episode) versus iteroparity (e.g. multiple reproductive sequels)^17^, programmed life span as compared to wear-and-tear aging processes^14,16^, weekly aging of colonial modules (blastogenesis)^22^, rejuvenation^23–26^, and the immortality of germ/somatic cell lines^27^. The above phenomena reflect the existence of spatial and seemingly stochastic age-mosaic modules within a genotype, the regulation of aging by an extreme regeneration power, and the replacement of somatic modules (sensu the disposable soma tenet^13,28^), all targeting a modular, colonial species that does not age according to the common aging phenomena in unitary organisms^14–16^. Considering the *disposable soma theory*, aging is considered as the inevitable outcome of decisions performed along the organism life span, all associated with the distribution of energy sources between the soma and the germ line. As long as the organism is sexually sterile, sources are allocated to growth, maintenance, repair, storage and defense; whereas sexual reproduction diverges the sources towards the germline, further imposing senescence^29–32^. While the above holds for unitary organisms that display germline sequestering, in colonial organisms that do not sequester their germline (such as *Botryllus*^33^), evolutionary trends may shift to biological statuses where the soma is not carefully maintained nor efficiently repaired (e.g.,^24,28,34^), somatic constituents that are replaced on a regular basis, and biological statuses that exhibit distinctive senescence occurrences^13^.

In *Botryllus,* most NF colonies present a single *Orshina* segment throughout their lives (Fig. 6, inner circle) and then they die. The fissioned colonies in FA and FB life history strategies (Fig. 6, outer circle) contain two or more *Orshina* segments, where at the end of each segment, rejuvenation may start off, leading to a new *Orshina* segment. The pivotal rejuvenation points (at *Orshina* phase δ) represent a distinct transition from colonial deterioration to growth and development that emerges after the completion of colonial fission events, which coincides with the death of ramets that do not surpass this critical turning point in astogeny. Therefore, ‘fission’ at the genet level may influence longevity. This is similar to mechanically fissioned annelids, where repeated fissions extended life spans^35^, fission augmented the growth in ramets of didemnid ascidians^36^, and elongated telomeres in the fissiparous starfish *Coscinasterias*^37^.

The literature on botryllid ascidians thoroughly discusses the astogenic phenomenon of blastogenesis (Fig. 1b), the weekly recurrent life and death events that develop at the modules level^11,12,27,38–41^. Basically, the *Orshina* segment described here is a newly elucidated life and death event that develops at the genet level, lasts an average of about three months on the average (containing approximately 13 blastogenic cycles), ends either with the death of the whole colony or with rejuvenation, and is manipulated by absence or existing fission events at the colony level, all of which reveal comparable and disparate properties when compared to blastogenesis (Table 1). A blastogenic cycle, in turn, operates at the level of zooids, lasts approximately one week, ends with the complete eradication of existing functional zooids and their replacement by a new developing set of primary buds (during the take-over phase) and fission occurs at the level of zooidal system (Table 1). However, the two phenomena, blastogenesis and *Orshina* rhythm, appear to be ‘programed’ by similar coding rules, sensu “As above so below, as below, so above” (attributed to *Hermes Trismegistus* in ‘Hermetic Corpus’).

**Table 1:** Comparative attributes for a blastogenic cycle and the *Orshina* segment.

Character	Astogenic agent	
	Blastogenesis	*Orshina* segment
Operation at the	Zooids level^5,43^	Colony level
Mean time of a single episode	About 7 days (in 20 °C)^20^	89 ± 45 days
From onset to termination	Zooids maturation (open siphons) to their death^43^	Dictated by RS, CV, size and fission statuses. Onset of an *Orshina* segment starts with male gonads, an increased CV and no. of zooids, while terminates with hermaphroditism, low no. of zooids, low CV statuses and complete fission events (when developed)
Phases	Four major stages, A-D^10,42^. Takeover of the colony with primary buds at the end of each cycle	Four major phases, α-δ. Colonial rejuvenation (no takeover) at pivotal state of death/live
Biomass (number of zooids) along one astogenic episode	Fixed, established at blastogenic stage A	Variable, characterized by a bell-like outline along each segment
Last phase of a segment/cycle	At the last day of the blastogenic cycle. Concludes with zooids death, their absorption and replacement by a new generation of zooids^44,45^	At the outskirts of segments. Concludes with either rejuvenation or colonial death
Death	At the zooids level. Occurring at the end of a cycle. Functional zooids commit a synchronized death through a massive apoptotic event^11,44^	At the colony level. Ramets may commit non-random death, in a synchronized fashion. Death is associated with the *Orshina* segment outskirts
Division/ fission	At the system level. A large system (usually 12 or more zooids) may divide into two or more systems^11^	At the genet level. A colony is fissioned into two or more ramets, in a synchronized fashion with the *Orshina* segment’s outskirts
Colors along the cycle	Zooids: Start/mid cycle: low pigmentation. End cycle: high pigmentation^46^	Colony: Start/mid cycle: low pigmentation. End cycle: high pigmentation (in accordance with CV scores along a segment)

The average life span of *Botryllus* colonies in our experiments (290 days^18^) is composed of 41 blastogenic cycles and 2.1 *Orshina* segments. *Botryllus schlosseri* genets thrive through rhythmic astogenic (*Orshina*) segments, each of which roughly lasts for three months that are most likely programmed by inherent and conserved genetics. Given optimal environmental conditions, a *Botryllus* colony, like *Orshina* the bird, is potentially able to live for long periods and rejuvenate in a rhythmic manner, a notion supported by the literature, where genets live for over 20 years^14^ or die through a programmed life span^16^. The current findings indicate that reproduction, life span, death, rejuvenation and fission events are, at least in part, scheduled processes along the constructed *Orshina* rhythm.

## Supplementary Information

Supplementary Figures.

Supplementary Tables.

## Data Availability

All data generated or analyzed during this study are included in this published article (and its supplementary information files).
