# Supplementary material for: The novel Orshina Rhythm in a colonial urochordate signifies the display of recurrent aging/rejuvenation sequels
Source: Sci Rep. 2023 Jun 16;13:9788. doi: 10.1038/s41598-023-36923-6 (PMC10276000; doi:10.1038/s41598-023-36923-6)

Supp. Fig 1. Individual graphs for 35 NF colonies studied from birth to death. Observations were made every 15±5 days. Three parameters were followed: number of zooids, RS and CV. X axis shows the timescale from birth to death. Left y-axis shows the number of zooids. Right y-axis shows either RS or CV. Green curves = number of zooids. Red curves = RS. Black curves = CV. Black vertical lines are *Orshina* borders that mark the segments. Numbers above segments show lengths (days) of segments. Missing numbers represent cases where borders could not be set. These segments were not added to statistical analyses.

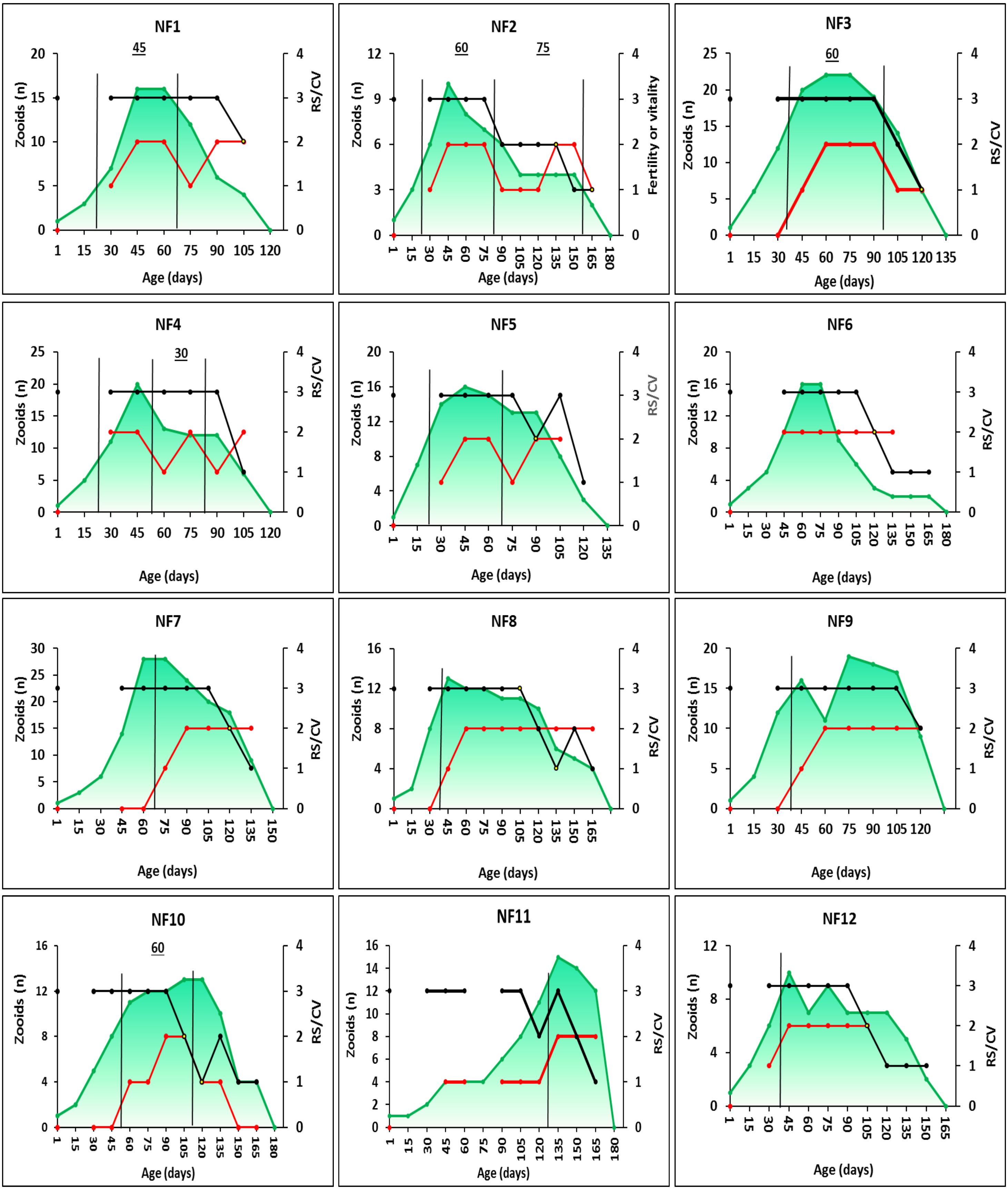

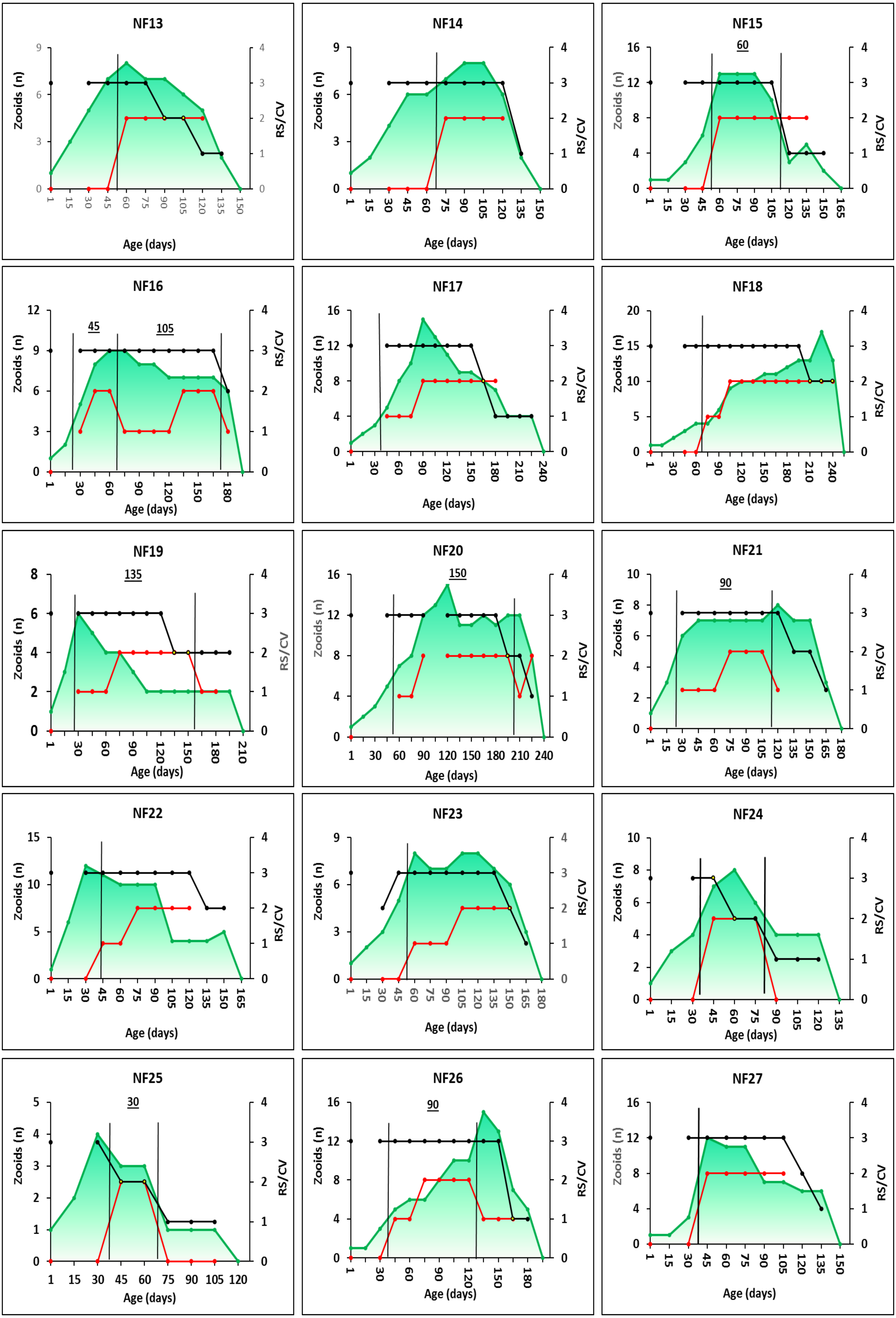

NF13
Zooids (n)
RS/CV
Age (days)
NF14
Zooids (n)
RS/CV
Age (days)
NF15
60
Zooids (n)
RS/CV
Age (days)
NF16
45
105
Zooids (n)
RS/CV
Age (days)
NF17
Zooids (n)
RS/CV
Age (days)
NF18
Zooids (n)
RS/CV
Age (days)
NF19
135
Zooids (n)
RS/CV
Age (days)
NF20
150
Zooids (n)
RS/CV
Age (days)
NF21
90
Zooids (n)
RS/CV
Age (days)
NF22
Zooids (n)
RS/CV
Age (days)
NF23
Zooids (n)
RS/CV
Age (days)
NF24
Zooids (n)
RS/CV
Age (days)
NF25
30
Zooids (n)
RS/CV
Age (days)
NF26
90
Zooids (n)
RS/CV
Age (days)
NF27
Zooids (n)
RS/CV
Age (days)

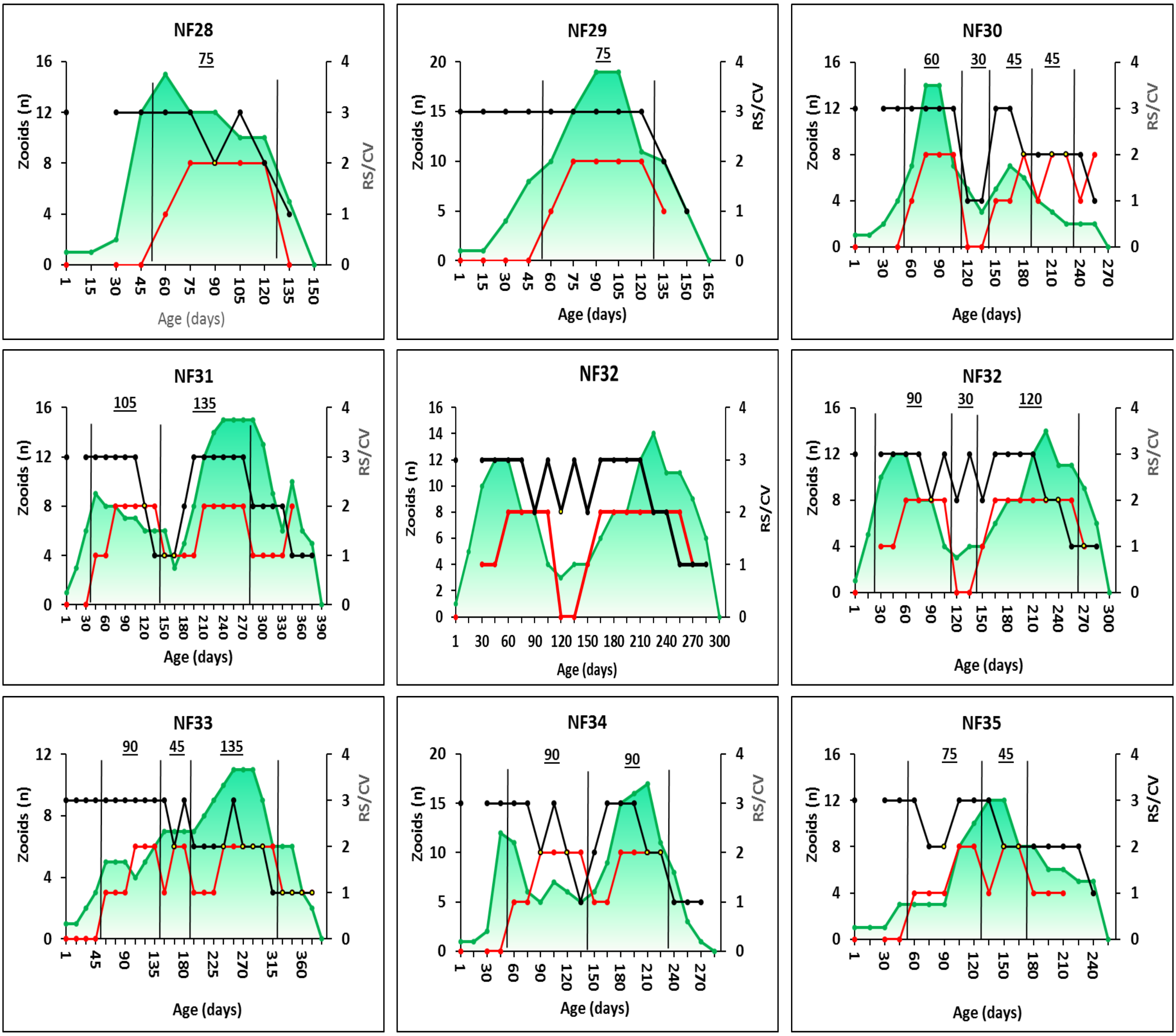
NF28
75
Zooids (n)
RS/CV
Age (days)
NF29
75
Zooids (n)
RS/CV
Age (days)
NF30
60
30
45
45
Zooids (n)
RS/CV
Age (days)
NF31
105
135
Zooids (n)
RS/CV
Age (days)
NF32
Zooids (n)
RS/CV
Age (days)
NF32
90
30
120
Zooids (n)
RS/CV
Age (days)
NF33
90
45
135
Zooids (n)
RS/CV
Age (days)
NF34
90
90
Zooids (n)
RS/CV
Age (days)
NF35
75
45
Zooids (n)
RS/CV
Age (days)

Supp. Fig 2. Individual graphs for 23 FA colonies studied from birth to death. Observations were made every 15±5 days. Three parameters were documented: number of zooids, RS and CV. X axis shows the timescale from birth to death. Left y-axis shows the number of zooids. Right y =axis shows either RS or CV. Green curves = number of zooids. Red curves = RS. Black curves = CV. Black vertical lines are *Orshina* borders that mark the segments. Numbers above segments show lengths (days) of segment. Fissions are marked with orange dashed lines. Deaths of ramets marked with red crosses. Missing numbers represent cases where borders could not be set. These segments were not added to statistical analyses.

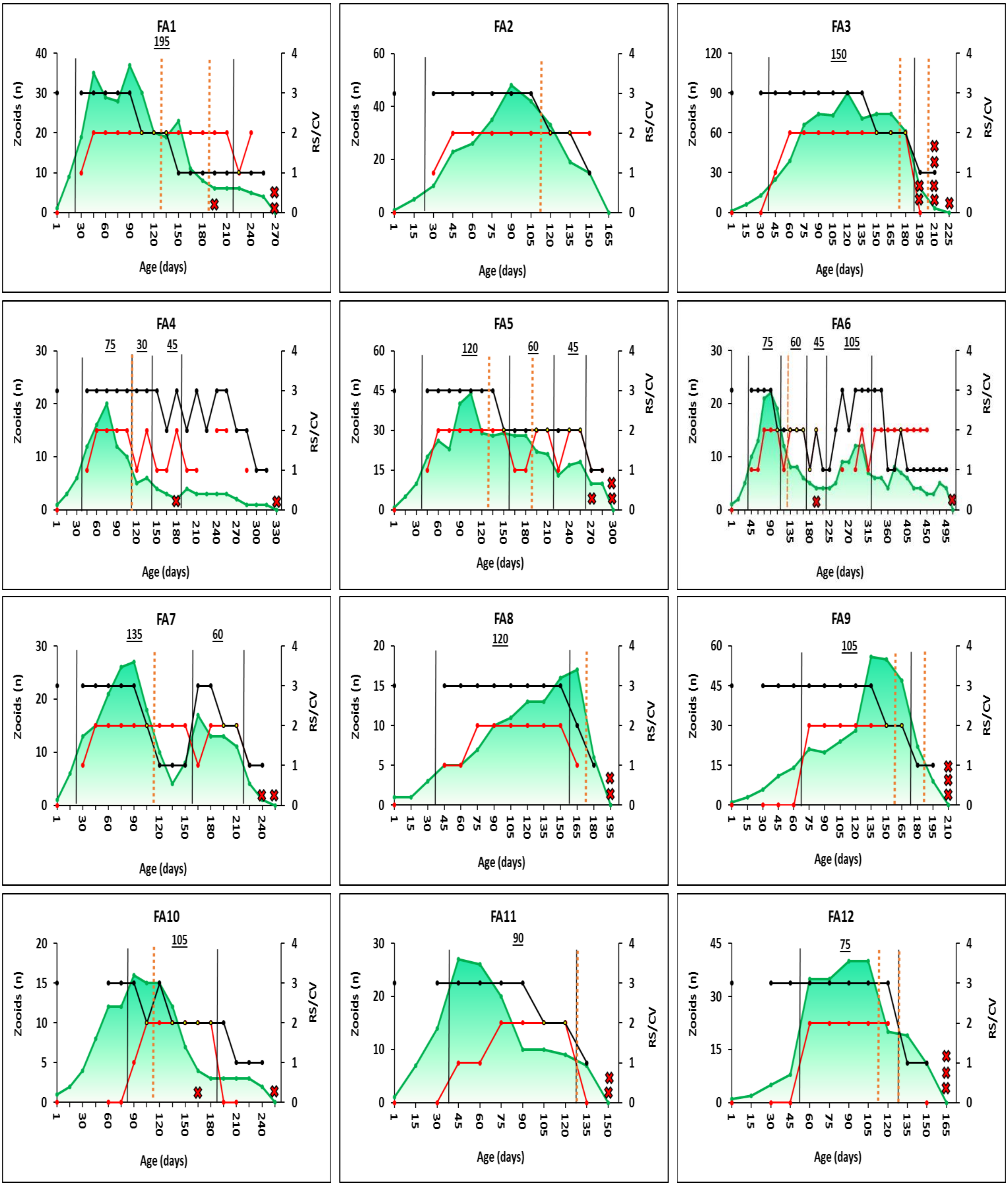

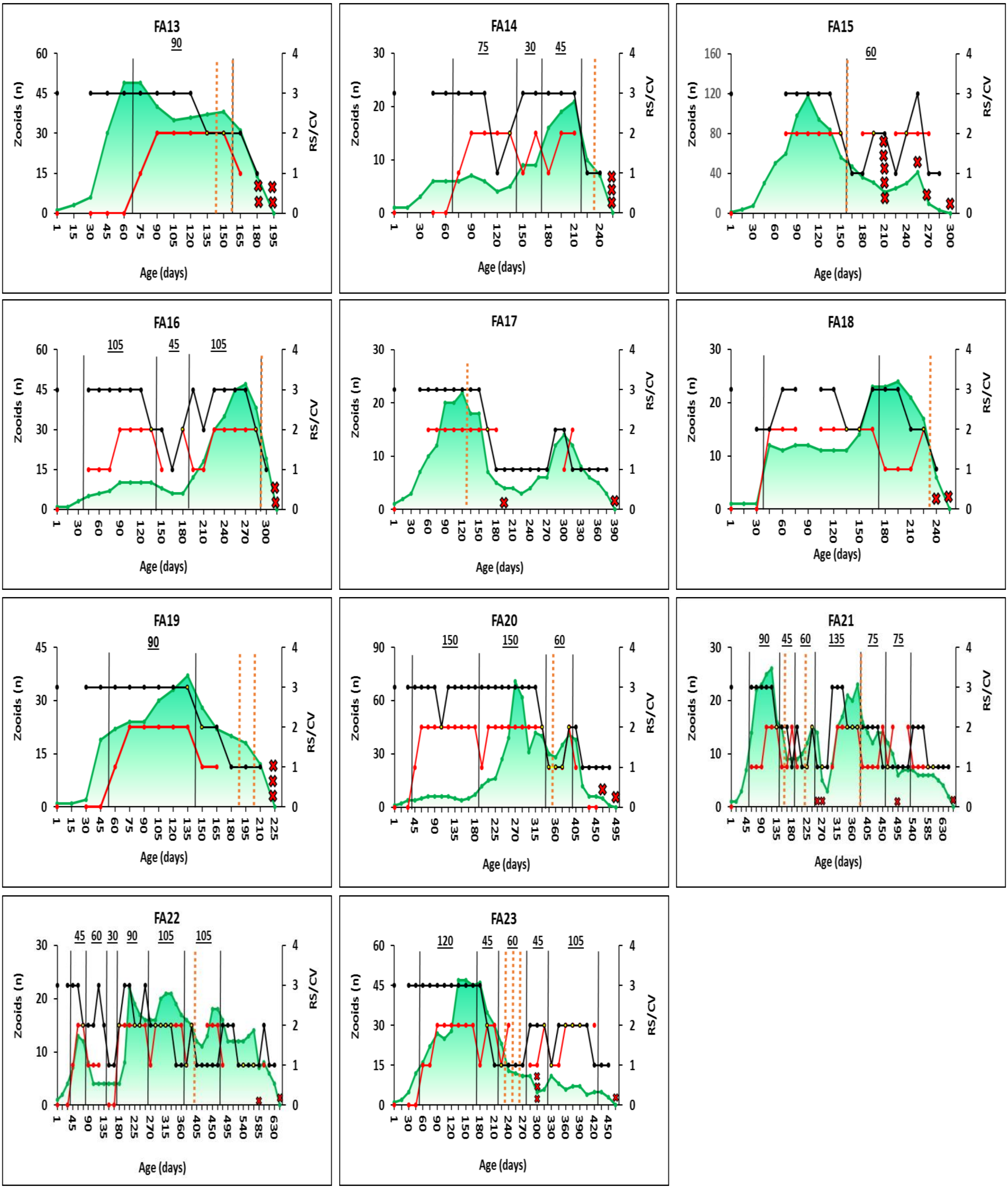
FA13
90
Zoids (n)
RS/CV
Age (days)
FA14
75
30
45
Zooids (n)
RS/CV
Age (days)
FA15
60
Zooids (n)
RS/CV
Age (days)
FA16
105
45
105
Zooids (n)
RS/CV
Age (days)
FA17
Zooids (n)
RS/CV
Age (days)
FA18
Zooids (n)
RS/CV
Age (days)
FA19
90
Zooids (n)
RS/CV
Age (days)
FA20
150
150
60
Zooids (n)
RS/CV
Age (days)
FA21
90
45
60
135
75
75
Zooids (n)
RS/CV
Age (days)
FA22
45
60
30
90
105
105
Zooids (n)
RS/CV
Age (days)
FA23
120
45
60
45
105
Zooids (n)
RS/CV
Age (days)

Supp. Fig 3. Individual graphs for 23 FB colonies studied from birth to death. Observations were made every 15±5 days. Three parameters were documented: number of zooids, RS and CV. X axis shows the timescale from birth to death. Left y-axis shows the number of zooids. Right y =axis shows either RS or CV. Green curves = number of zooids. Red curves = RS. Black curves = CV. Black vertical lines are *Orshina* borders that mark the segments. Numbers above segments show lengths (days) of segment. Fissions are marked with orange dashed lines. Deaths of ramets marked with red crosses. Missing numbers represent cases where borders could not be set. These segments were not added to statistical analyses.

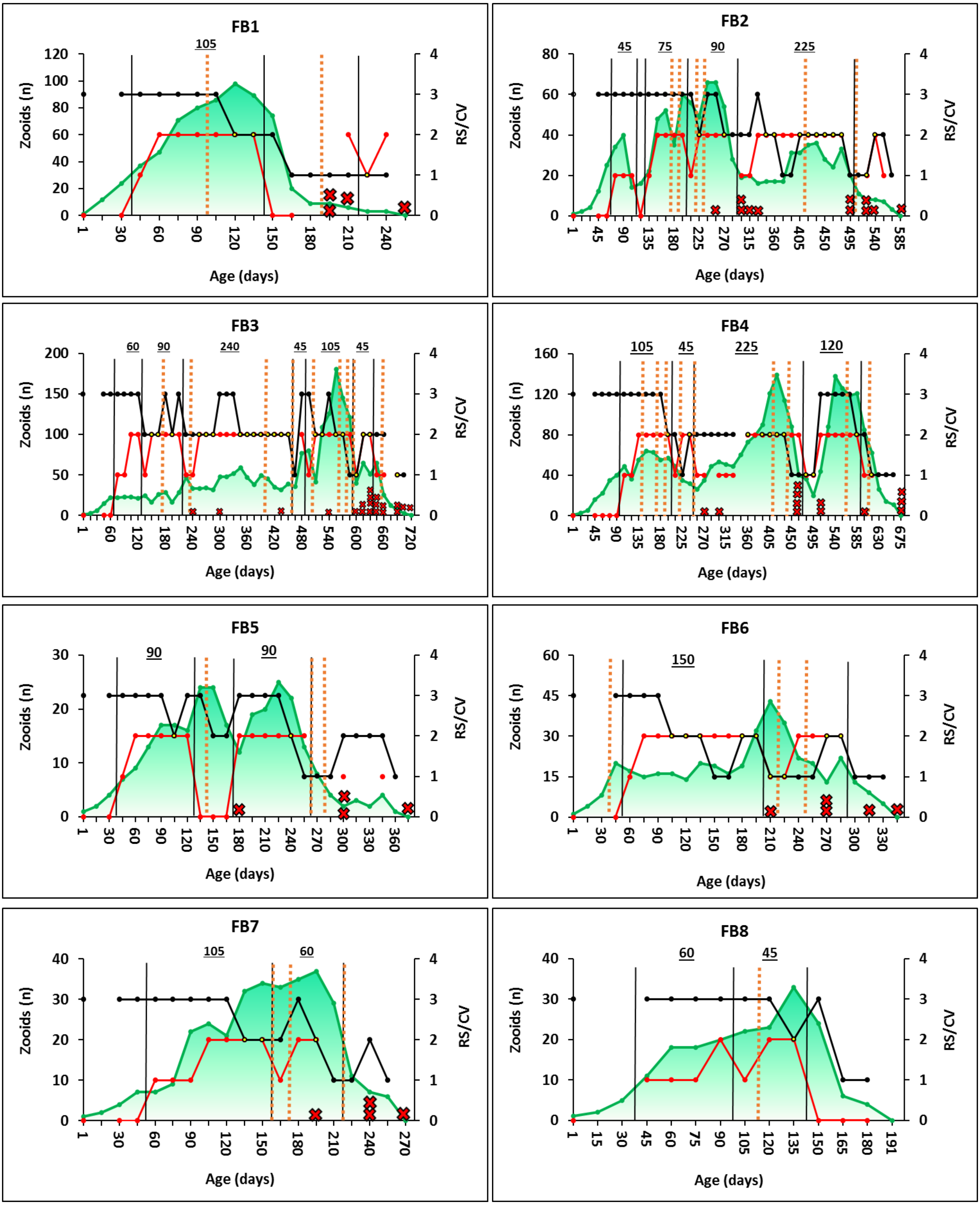

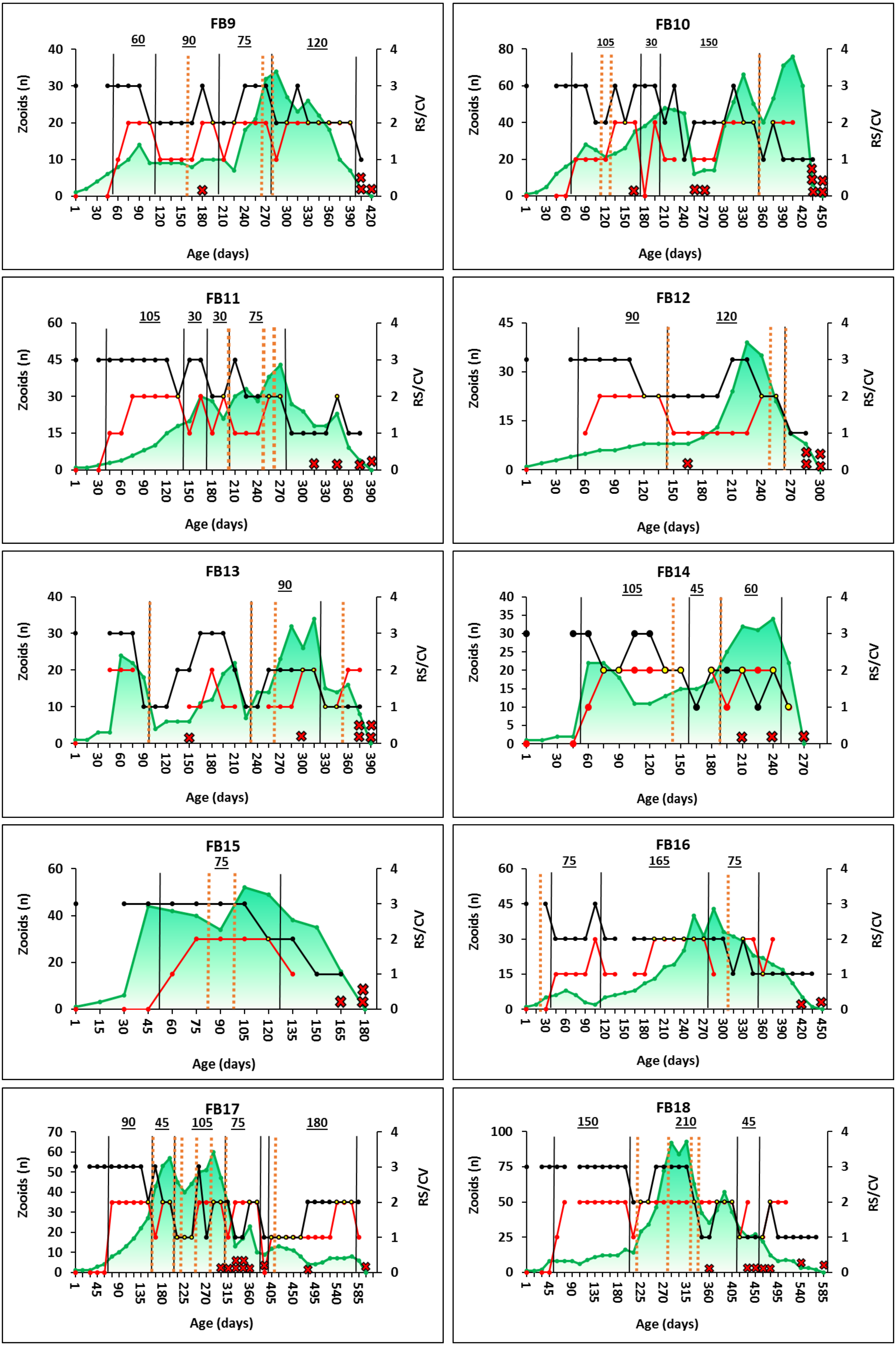

FB9
60
90
75
120
Zooids (n)
RS/CV
Age (days)
FB10
105
30
150
Zooids (n)
RS/CV
Age (days)
FB11
105
30
30
75
Zooids (n)
RS/CV
Age (days)
FB12
90
120
Zooids (n)
RS/CV
Age (days)
FB13
90
Zooids (n)
RS/CV
Age (days)
FB14
105
45
60
Zooids (n)
RS/CV
Age (days)
FB15
75
Zooids (n)
RS/CV
Age (days)
FB16
75
165
75
Zooids (n)
RS/CV
Age (days)
FB17
90
45
105
75
180
Zooids (n)
RS/CV
Age (days)
FB18
150
210
45
Zooids (n)
RS/CV
Age (days)

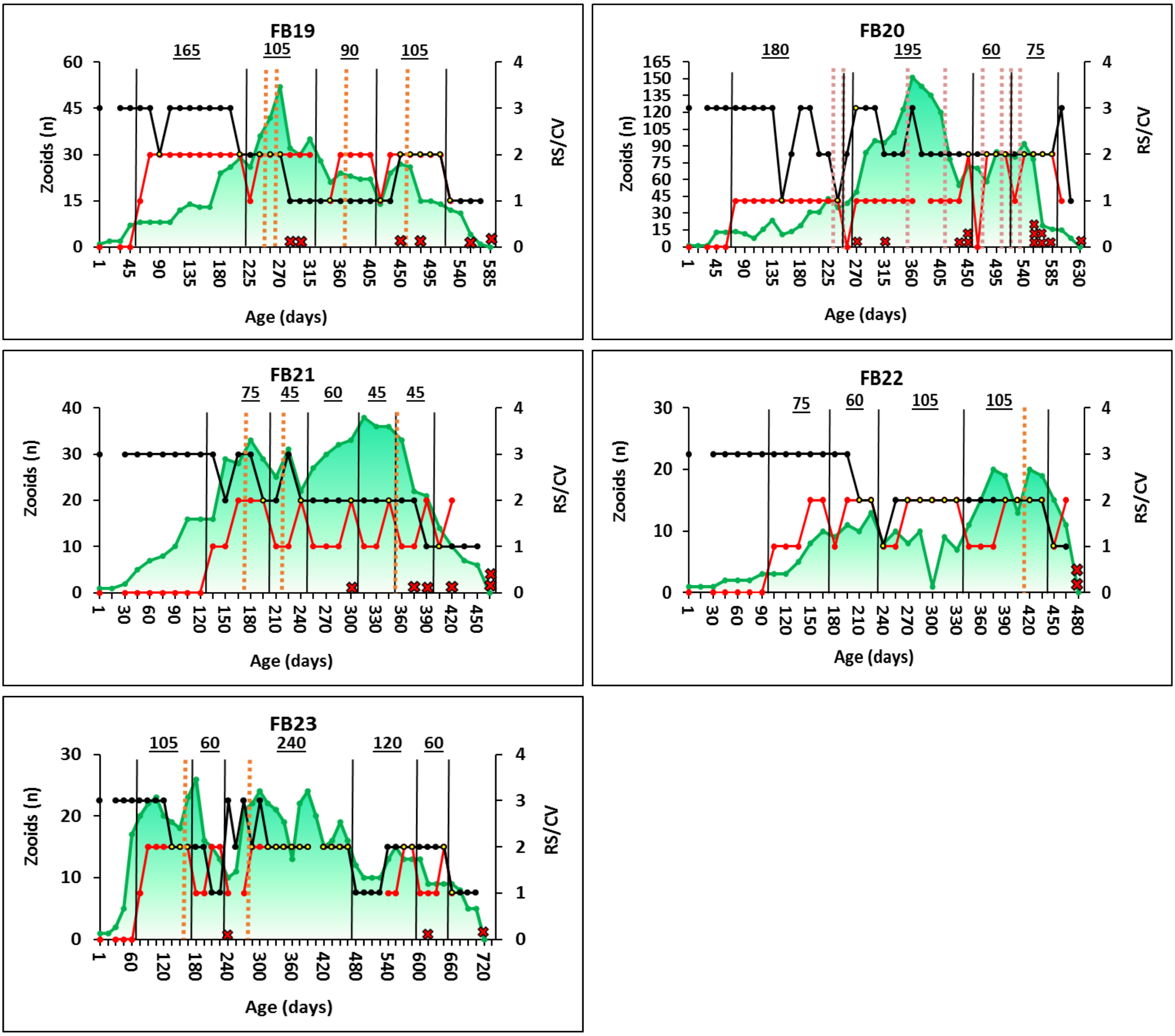
FB19
165
105
90
105
Zooids (n)
RS/CV
Age (days)
FB20
180
195
60
75
Zooids (n)
RS/CV
Age (days)
FB21
75
45
60
45
45
Zooids (n)
RS/CV
Age (days)
FB22
75
60
105
105
Zooids (n)
RS/CV
Age (days)
FB23
105
60
240
120
60
Zooids (n)
RS/CV
Age (days)

Suppl Fig. 4. Colonial Vigorousness Score (CV) Represented by Visual Assessment of Colonies. The visual representation of the Colonial Vigorousness Score (CV) showcase colonies at different CV statuses. The CV scale ranges from 1 to 3, with higher scores indicating greater vitality and healthier colonies. CV = 3: Maximal Vitality. In this state, colonies exhibit optimal vigor and vitality. Zooids display a remarkable level of uniformity and synchronization. They possess identical shapes and minimal pigmentation. The tunic, covering the colony, is transparent and intact. Furthermore, the ampullae are inflated and elongated. CV = 2: Fair Vitality. Colonies exhibit a moderately healthy state. Zooids display increased pigmentation, and their sizes may exhibit slight variations. The transparency of the tunic is reduced, giving it a cloudy appearance. Additionally, the ampullae may change in size, and become dislocated from the edges of the tunic. CV = 1: Lowest Vitality. Zooids within the colony exhibit significant pigmentation. The tunic becomes cloudier. The ampullae display abnormal sizes and show a considerable dislocation from the edge of the tunic.

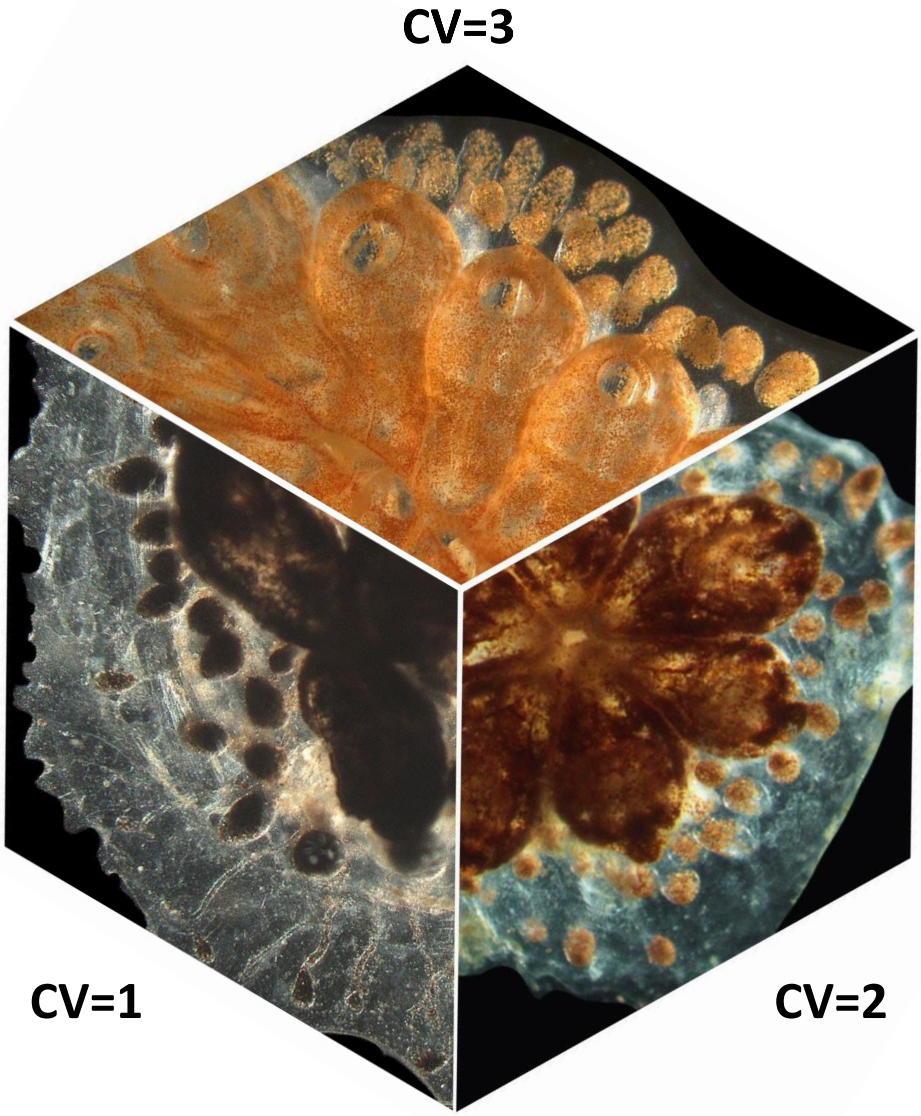

Supplement: Supplementary file 1 — Supplementary Figures. [file 41598_2023_36923_MOESM1_ESM.pdf]
